# The Finger Nails

**Published:** 1891-04

**Authors:** 


### THE FINGER NAILS.

There is a common belief that the finger nails are poisonous, which idea is natural enough, considering the fact that scratches made by them are generally quite irritable and much inclined to unusual inflammation. The reasoning is erroneous, however, for, as far as is known, the nails themselves do not have any poisonous properties. The trouble excited by them is due to the foreign deposits under them. In other words, if one keeps his finger nails clean, scratches caused by them will be no more irritable than those produced by any like instrument that is considered innocent. The results of the examinations made in Vienna show that it is more important that the finger nails be kept clean than is supposed. Seventy-eight were made and there were found thirty kinds of micrococci, eighteen different bacilli and three kinds of sarcenæ; besides, common mold spores were present in many instances. It would seem from this that the spaces under the finger nails were favorable hiding places for minute organisms which are more or less prejudicial to health, and that therein lies the poisonous element attributed to the nails. Furthermore, that cleanliness of the nails is a very important essential. It is not sufficient to use merely a knife blade, but at the toilet a nail brush and plenty of soap and water should be called into service. Surgeons long ago learned that deposits under the nails were a menace, and that through them wounds were easily poisoned. This led to extreme care in the matter of personal cleanliness on their own part and on the part of all their assistants. Before an operation is performed all who touch the patient or the instruments which are to be used must first clean their hands thoroughly with soap and water, being especially careful to have the spaces under the nails absolutely clean ; then the hands are put into disinfectant solutions.

